# Antenatal care data sources and their policy and planning implications: a Palestinian example using the Lives Saved Tool

**DOI:** 10.1186/s12889-019-6427-8

**Published:** 2019-01-30

**Authors:** Ingrid K. Friberg, Mahima Venkateswaran, Buthaina Ghanem, J. Frederik Frøen

**Affiliations:** 1 Global Health Cluster, Division for Health Services, Norwegian Institute of Public Health, P.O.Box 222 Skøyen, N-0213 Oslo, Norway; 2 Centre for Intervention Science in Maternal and Child Health (CISMAC), University of Bergen, Bergen, Norway; 3 World Health Organization, Palestinian National Institute of Public Health, Al Bireh P.O.Box 4284, Ramallah, Palestinian Territory

**Keywords:** Lives Saved Tool (LiST), Antenatal care indicators, Priority setting in maternal and child health, Data for policy-making

## Abstract

**Background:**

Policy making in healthcare requires reliable and local data. Different sources of coverage data for health interventions can be utilized to populate the Lives Saved Tool (LiST), a commonly used policy-planning tool for women and children’s health. We have evaluated four existing sources of antenatal care data in Palestine to discuss the implications of their use in LiST.

**Methods:**

We identified all intervention coverage and health status indicators around the antenatal period that could be used to populate LiST. These indicators were calculated from 1) routine reported data, 2) a Multiple Indicator Cluster Survey (MICS), 3) paper-based antenatal records and 4) the eRegistry (an electronic health information system) for public clinics in the West Bank, Palestine for the most recent year available. We scaled coverage of each indicator to 90%, in public clinics only, and compared this to a no-change scenario for a seven-year period.

**Results:**

Eight intervention coverage and health status indicators needed to populate the antenatal section of LiST could be calculated from both paper-based antenatal records and the eRegistry. Only two could be calculated from routine reports and three from a national survey. Maternal lives saved over seven years ranged from 5 to 39, with percent reduction in the maternal mortality ratio (MMR) ranging from 1 to 6%. Pre-eclampsia management accounted for 25 to 100% of these lives saved.

**Conclusions:**

The choice of data source for antenatal indicators will affect policy-based decisions when used to populate LiST. Although all data sources have their purpose, clinical data collected directly in an electronic registry during antenatal contacts may provide the most reliable and complete data to populate currently unavailable but needed indicators around specific antenatal care interventions.

**Electronic supplementary material:**

The online version of this article (10.1186/s12889-019-6427-8) contains supplementary material, which is available to authorized users.

## Background

Setting effective and appropriate national, sub-national or sector-wide policies is a complex endeavor for health systems everywhere. Investigations of priority setting at national levels have demonstrated a high degree of similarity; critically, a unified understanding of the importance of the health problem is vital [1, 2]. A common complaint among policy makers is the inability to trust the evidence and data, especially when international and local numbers differ [3]. As a result, consistent sources of high quality and trustworthy data, tailored to the local context to inform planning processes, have proven to be a clear gap [4].

High quality data can be used at different points in the policy planning cycle, including for informing discussions as well as projecting the impacts of potential decisions, both of which are commonplace activities. The Lives Saved Tool (LiST) is a policy planning tool which utilizes information on the current health status of a country to project the health (mortality) implications of implementing specific health interventions for women or children [5]. LiST has been used for over ten years for evaluation, advocacy and strategic planning [6], across a wide variety of settings [7, 8]. An unsurprising criticism of LiST is the quality of data available to populate it [9] – in many instances, significant assumptions and estimations are required given the lack of primary data [10]. For any modelling tool, as for any policy setting process, high quality data is required to ensure that the results are accurate enough for usability [9].

LiST requires health status indicators (such as mortality and morbidity), effectiveness data (impact of interventions on health status), and coverage indicators (levels of utilization of health interventions). The coverage indicators required to populate LiST come from a variety of sources, including national statistics, household surveys, facility surveys and research studies, and are less amenable to global evaluation and summarizing due to variability in the implementation of many of these interventions. Few countries have routine high quality data on effective coverage (proportion of those getting an intervention among those in need) for assessing all aspects of their health system within the LiST structure. The frequency and quality of routinely reported data from health systems vary by topic and country, leaving alternative sources of data necessary. Many countries rely on externally funded, population-based surveys such as UNICEF’s Multiple Indicator Cluster Survey (MICS) [11] and the Demographic and Health Surveys (DHS) [12] to collect service related data by asking women to remember the care received during their most recent pregnancy [13], often up to 2–5 years in the past.

The ever-expanding arena of information technology and digital registries has the potential to improve data availability around interventions delivered during antenatal care, childbirth and the postpartum period [13]. ‘eRegistries’ are electronic registries used at the point of care for recording health services delivered [14]. They are specifically designed to facilitate implementation of several digital health interventions such as: decision support tools, and audit and feedback (to aid health care workers in providing quality care); tailored behavior change communication text messages (to encourage women to attend care); and reporting (to provide aggregate data for health system managers and policy makers). An eRegistry for antenatal, postpartum and newborn care has been rolled out in primary health care clinics in the public sector in Palestine as part of a national implementation [15].

The validity of LiST outputs and results is closely linked to the kind of data that is input [5]. However, few studies have assessed the nature and magnitude of consequences to LiST results when using different sources of data. Users of LiST should be aware of such consequences to make informed decisions about intervention effectiveness when considering scale-up. Our objective was to model the scale up of antenatal care interventions in LiST, using all available data sources in Palestine – routine data, survey results, extracted medical records and the eRegistry, to explore how the results might vary, and the implications of using these varied sources to make decisions.

## Methods

### Study design

This secondary data analysis utilized multiple sources of health information for modeling mortality and morbidity impacts of scaling up coverage of routine health interventions delivered during the antenatal period in the Lives Saved Tool.

### Indicators for the Lives Saved Tool

We identified all coverage and health status indicators needed to fully model antenatal care in the Lives Saved Tool (LiST). For each of those indicators, we then selected those that were: 1) relevant to the population in the West Bank and 2) available in any of the known data sources. Malaria, HIV/AIDS and syphilis indicators were not considered as these are not common health issues in the Palestinian population. Neither calcium supplementation nor balanced energy supplementation were part of the national guidelines recommended for the public health system in the West Bank, and were not considered. Although mortality data were also needed, they were not extracted from any of the data sources; identical default mortality data from the World Health Organization and LiST were used for all analyses.

### Data sources

#### Routine reporting data

Routine data for 2016, as reported by clinical workers, were available for the West Bank, including number of women attending antenatal care at public vs. other centers [16].

#### Population based survey data

The most recent population-based survey in Palestine which included antenatal care data was the 2014 Multiple Indicator Cluster Survey, published in 2015 [17]. As part of this population-weighted survey, a nationally representative sample of women were asked about utilization of antenatal care, including the location and type of tests performed for pregnancies completed within the past 2 years. Using the published weights, we calculated the proportion of women attending antenatal care at public facilities. All data from live births in the West Bank were included in this analysis; no available records were excluded for any reason.

#### Data from antenatal records

##### Paper-based records

In preparation for the national implementation of the eRegistry in Palestine, all antenatal records from 17 primary healthcare clinics in five districts in the West Bank were extracted for the year 2015, for a total of 1369 pregnancies [18]. The clinics were randomly selected to be representative of the districts where the first phase of the national implementation would take place. There were no individual inclusion or exclusion criteria; records from all pregnant women were extracted. Clinical data were extracted from the paper-based records and entered into electronic data entry forms that were identical to the data entry forms of the eRegistry (see below). Quality checks of data entry were carried out; 10% of all paper-based records were entered twice by the data extractors.

##### eRegistry data

Care providers at public antenatal clinics in 76 facilities in five districts in the West Bank directly entered antenatal care records into an eRegistry throughout the year 2017. These clinics include all the primary health care clinics in the same five districts as the paper-based record extraction. There were no individual inclusion or exclusion criteria; records from all pregnancies entered into the eRegistry were included in the analysis. Records with no valid data entered were excluded. We used this data for all pregnant women registered on or after January 1, 2017 and passed 44 weeks of gestation as of 30th of April 2018.

##### Differences between the paper and eRegistry records

Although the paper extraction and the eRegistry were designed to be identical, differences did exist; specifically, they contained notably different data on iron-folate supplementation (Table 1). In the paper records, a single data point recorded whether iron-folate supplements were given. In the eRegistry, integrated clinical decision support reminded the care provider of the specific dose of iron-folate required, and care providers documented whether or not the suggested management was performed.

**Table 1:** Definition and calculation of antenatal care indicators for the Lives Saved Tool

	LiST Indicators	Reporting	Household Survey	Antenatal Records	
		Routine (2016)	MICS (2014)	Paper (2016)	eRegistry (2017)
Coverage	% of women with diabetes with appropriate management	Cases of diabetes referred / (pregnant women registered X diabetes incidence‡)	Indirect calculation; Kanyangarara method†	% of women screened for diabetes at the correct time in pregnancy, and referred if needed; see Fig. 1	
	% of women with hypertensive disorders in pregnancy with appropriate management	NA	Indirect calculation; Kanyangarara method†	% of women screened for hypertension at the correct time in pregnancy, and referred to the high-risk clinic if needed	
	% of women with appropriate tetanus toxoid vaccination	NA	NA	% of women who are vaccinated according to guidelines	% of women who are vaccinated according to guidelines at enrollments AND given a booster if unknown/unimmunized
	% of women with pre-eclampsia who have been referred	Cases of pre-eclampsia referred / (pregnant women registered X pre-eclampsia incidence‡)	Indirect calculation; Kanyangarara method†	% of pregnant women screened for pre-eclampsia at the correct time in pregnancy and referred to the hospital, if needed	
	% of pregnant women with iron and folic acid supplements	NA	NA	% of women given iron and folic acid supplements	% of non-anemic women given routine iron supplements and % of anemic women treated
Health status	% of pregnant women with anemia	NA	NA	% of pregnant women with a hemoglobin value of < 11 g/dl or less at any point during the pregnancy	
	% of women with low body mass index	NA	NA	% of women with body mass index < 18.5 at a booking visit	% of women with body mass index < 18.5 at a booking visit
	% of women with severe anemia	NA	NA	% of women with a hemoglobin level of < 7 g/dl or less ever	

*NA* Not available; †Additional File 1 for details. ‡Diabetes and pre-eclampsia incidence calculated from paper-based antenatal records

##### Calculation of LiST indicators from paper-based and eRegistry antenatal record data

**Fig. 1:**
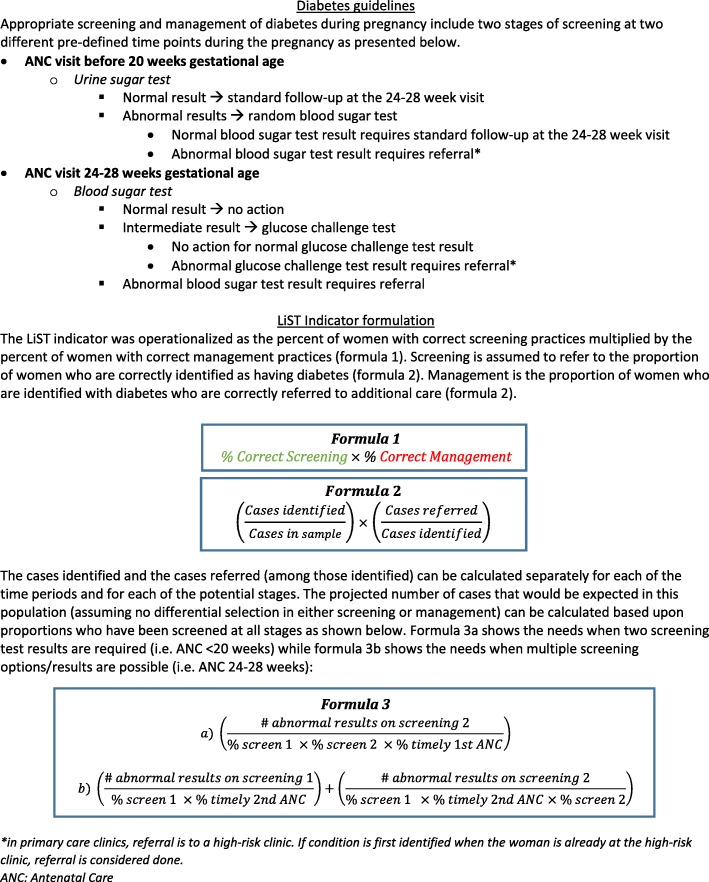
Conversion of diabetes guidelines in Palestine into an indicator for the Lives Saved Tool (LiST)

For LiST analyses, management indicators require data on 1) the proportion of women eligible for screening (including seeking care) who were screened correctly and at the correct time, and 2) the proportion of those identified who were correctly managed, among those that had a positive screening test (Fig. 1). This reflects the proportion of women who truly had a condition and were correctly managed of those that attended care at public facilities (Fig. 1).

**Fig. 2:**
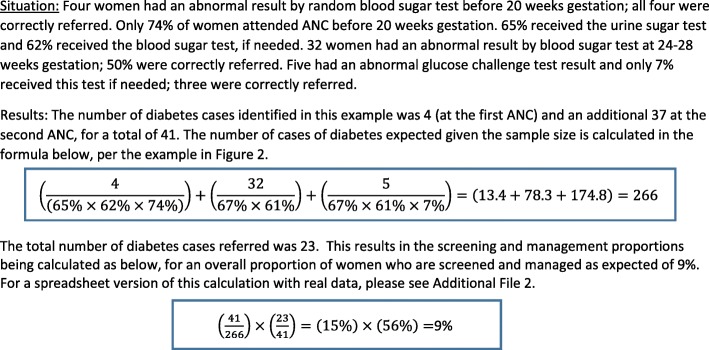
Worked example of converting diabetes screening and management practices into indicators for Lives Saved Tool (LiST)

In the West Bank, diabetes screening consists of urine sugar testing of all pregnant women at the booking antenatal visit, a blood sugar test at 24–28 gestational weeks for those not already positive, and a glucose challenge test based on blood sugar test results (Fig. 1). For women with a result greater than 140 mg/dl on the glucose challenge test, correct management is referral. Hypertension screening requires serial blood pressure measurement at all antenatal care visits. For mild hypertension, recommended management includes urine protein testing. Screening for pre-eclampsia requires a urine protein test following measurement of hypertension after 20 weeks gestation. Referral is the recommended management for women with chronic hypertension, moderate or severe gestational hypertension, hypertension with proteinuria or symptoms of preeclampsia. We assumed correct management for all correct referrals regardless of whether women sought that additional care at the referral facility or not. We also assumed equitable screening and management of all pregnant women irrespective of health or socio-economic characteristics. Figure 2 contains a worked example of how the indicator for diabetes management was calculated, based on the construction in Fig. 1. Additional File 2 displays the detailed calculations. All data are available upon request.

For indicators unable to be calculated directly from the data sources, we utilized the Kanyangarara method [19], developed specifically to utilize distal determinants to predict coverage for LiST.

### Lives Saved Tool analyses

LiST (version 5.71; Avenir Health) predicts the number of deaths and anemia cases that would have occurred under a given population and health scenario, combined with coverage of health interventions and how they change over time [20]. We compared two national level scenarios: 1) a steady state scenario from 2017 to 2025 and 2) a scenario where coverage of antenatal care interventions increased to 90% from baseline in public facilities only (with no change in other facilities) in 2018, and then remained at a steady state through 2025. The primary result is the difference in the number of deaths and anemia cases during 2018–2025 between the two scenarios. All sources reported data from slightly different time periods, and to mimic a typical situation, we applied the most recently available data to the year 2017. We assumed that the quality of care delivered to women attending both public and other facilities was constant.

The proportion of women attending antenatal care in public facilities for the MICS analysis came directly from the survey itself. For LiST analyses using the other three data sources, we used the routinely reported estimates of the proportion of women attending public facilities. Over time, we assumed no change in the proportion of women attending public vs. other clinics nor in the quality of care provided at other clinics.

## Results

### Data to indicators

The coverage and health status indicators available for input in LiST are presented in Table 1 along with the exact definitions and calculations for each data source. The number of women managed with diabetes or pre-eclampsia were available from the routine data, although incidence values were not available. To allow this analysis to proceed, we required a source of incidence, which we derived from our medical record (paper-based) review. Indicators of management of diabetes, hypertensive disorders and pre-eclampsia were not available from the MICS. They were indirectly calculated using the Kanyangarara method [19] (Additional File 1). Although available in some MICS, the Palestinian survey did not include indicators related to tetanus vaccination or iron supplementation. Although data on symphysis-fundal height measurement were available in both the paper-based records and eRegistry data, management data were not; identification and management of fetal growth restriction was not calculated for any source. Data from paper-based records and the eRegistry included all pregnancy indicators of interest. Women with moderate or severe hypertension or potential pre-eclampsia are referred to hospitals and do not return to the primary care clinics for ANC management; as a result, the proportion of women correctly identified and managed with pre-eclampsia may be incomplete. In addition, the amount of missing data for tetanus toxoid vaccination was notably different with 42% missing in paper records and only 7% missing in the eRegistry, although missing data proportions were much more similar in the two data sources for other indicators.

Five coverage indicators and three health status indicators for the West Bank could be calculated from the four sources of antenatal care data (Table 2). The routinely reported data populated two coverage indicators and none of the health status indicators, while the MICS data could directly populate none of the coverage or health status indicators. The MICS data could be used to indirectly calculate three of the coverage indicators. Data from paper-based antenatal records and the eRegistry were used to calculate all five of the coverage indicators and all three of the health status indicators.

**Table 2:** Summary of indicator availability by source

		Routine Data	MICS	Paper records	eRegistry
Coverage	Directly	2/5	0/5	5/5	5/5
	Indirectly*	0/5	3/5	0/5	0/5
	All	2/5	3/5	5/5	5/5
Health status		0/3	0/3	3/3	3/3

*Using the Kanyangarara method [19]

### LiST analysis

Table 3 summarizes the baseline and target inputs to a national level LiST analysis with coverage of appropriate care in public West Bank clinics increased to 90%, assuming no change in the proportion of women attending public facilities and no change in quality of care provided at other facilities. When we used routinely reported data or MICS data as the source for LiST analyses, increasing coverage would lead to no newborn deaths or anemia cases being averted (Table 4). Using routinely reported data, the LiST analysis estimated that 16 maternal deaths and 239 stillbirths would be averted. Using MICS data, LiST suggested that far fewer maternal deaths and stillbirths would be averted (Table 4). In contrast, the LiST analysis using individual level data from both the paper-based antenatal care records or the eRegistry led to comparable estimates of more maternal deaths potentially being averted, and that improving the quality of care in Palestine would also avert a number of newborn deaths. While LiST analyses based on routine data and MICS would be unable to identify a reduction in anemia cases by improving anemia prevention, both sources of individual level data suggested significant gains from better prevention.

**Table 3:** National level Indicators from all sources used as inputs in the LiST analysis

		Reporting	Survey	Antenatal Records	
Analysis	Indicators	Routine (2016)	MICS (2014)	Paper (2016)	eRegistry (2017)
National baseline (applied to 2017)	% of all pregnant women who have completed the appropriate tetanus toxoid vaccination schedule	NA	NA	85.4	92.1
	% of pregnant women taking the appropriate iron or folate supplementation	NA	NA	90.3	64.4
	% of women with hypertensive disorders in pregnancy who are correctly managed	NA	68.9†	15‡	35‡
	% of women with diabetes with appropriate case management	71.9	35.1†	7‡	10‡
	% of women with pre-eclampsia during pregnancy who are correctly managed	51.7	72.9†	11‡	14‡
	Anemia	27*	27*	37.3	37.7
	Severe anemia	0.272*	0.272*	0	0.1
	BMI	3.1*	3.1*	2.8	4.4
National target assuming 90% coverage in public sector (applied to 2018–2025)	% of all pregnant women who have completed the appropriate tetanus toxoid vaccination schedule	NA	NA	92,0	95,7
	% of pregnant women taking the appropriate iron or folate supplementation	NA	NA	94.7	80.6
	% of women with hypertensive disorders in pregnancy who are correctly managed	NA	75.5†	53.6	64.5
	% of women with diabetes with appropriate case management	84.7	47.3†	49.2	50.9
	% of women with pre-eclampsia during pregnancy who are correctly managed	73.6	74.5†	51.4	50.9
	Anemia	27.2*	27.2*	37.3	37.7
	Severe anemia	0.272*	0.272*	0	0.1
	Body mass index (BMI)	3.1*	3.1*	2.8	4.4

*LiST defaults: Finucane 2011 [28], Stevens 2013 [29]; †Using the Kanyangarara method [19] ‡See Additional File 2 for details

**Table 4:** Morbidity and mortality results

		Routine Data	MICS	Paper records	eRegistry
Morbidity & mortality	Maternal lives saved	16	5	35	39
	Newborn lives saved	0	0	49	39
	Stillbirths averted	239	45	285	270
	Maternal anemia cases averted	0	0	16,444	42,064
Interventions averting mortality and morbidity	Maternal	Pre-eclampsia management (100%)	Hypertensive disease management (75%); Pre-eclampsia management (25%)	Hypertensive disease management (41%); Pre-eclampsia management (59%)	Hypertensive disease management (45%); Pre-eclampsia management (55%)
	Newborn	–	–	Tetanus toxoid (100%)	Tetanus toxoid (100%)
	Stillbirth	Pre-eclampsia management (84%); diabetes management (16%)	Pre-eclampsia management (52%); diabetes management (48%)	Pre-eclampsia management (83%); diabetes management (17%)	Pre-eclampsia management (82%); diabetes management (18%)
	Anemia	–	–	Iron Folate (100%)	Iron Folate (100%)
Rates, ratios, percentages	Maternal Mortality Ratio (2017/2025) % change	46/44 3%	46/45 1%	46/43 6%	46/43 6%
	Neonatal Mortality Rate (2017/2025) % change	11/11 0%	11/11 0%	11/11 < 1%	11/11 < 1%
	Stillbirth Rate (2017/2025) % change	7/7 2%	7/7 < 1%	7/7 3%	7/7 3%
	Pregnant women with anemia (%) (2017/2025) % change	27/27 0%	27/27 0%	37/36 3%	38/35 8%

The specific interventions resulting in these deaths being averted were similar across data sources, with tetanus toxoid preventing all newborn deaths and iron folate supplementation preventing all anemia morbidity. The lack of data on hypertension management in the routine data resulted in all deaths being averted by pre-eclampsia management, while only 25% were prevented by pre-eclampsia management using the MICS data. Both data from paper-based records and the eRegistry suggested a similar proportion of maternal deaths being averted by pre-eclampsia management and hypertensive disorders management. Stillbirths were predominantly averted by pre-eclampsia management with a varying proportion averted due to diabetes management, based on the source utilized (Table 4).

## Discussion

Data for decision-making is a common cry in public arenas. However, not all data are the same, and the implications of using the various alternative data sources available can be significant, especially when multiple choices exist, such as in the case of Palestine. Selection of data source can be even more critical when used as inputs into a formal analytic framework, as many policy makers do not see the raw data but only the results of the processing, assumptions, estimates, and analysis. Global agencies and research teams publish consensus estimates of mortality with uncertainty bounds, but estimates of health intervention coverage show more variability and are less widely available in general. The availability of new sources of local and timely data and indicators is likely to increase as countries shift towards digital data and case-based collection methods. The evaluation of these new data sources is critical to assess their potential for improving the care being delivered and to appropriately inform planning processes.

In this analysis, the four data sources yielded notably different results when utilized in LiST. The maternal deaths averted ranged from 5 to 39, or a reduction of maternal mortality from 1 to 6%. At the same time, the composition of interventions to save these lives varied from 100% for pre-eclampsia management to 75% for hypertensive disorders management. These differences would likely result in different policy and practice decisions being taken. Similar, but less dramatic differences could be seen in newborn, stillbirth, and anemia results using the different data sources. Although the absolute differences were relatively small in this particular context, they would be magnified greatly in countries and settings with higher mortality and morbidity rates, or if interventions beyond antenatal care were included.

The power of the Lives Saved Tool can be maximized when data of better quality and quantity are available to populate it. However, in most country settings, several data points are not directly available in either routine reports or household surveys. Drawing data directly from clinical records allows for a more complete and complex picture of antenatal care and covers almost all data needs. Many surveys, such as the MICS, only include data from live-births [17], thus excluding data on women who experienced stillbirths or miscarriages and their potentially complicated pregnancies. Another aspect of clinical data, not present in most survey or routine data sources, is the longitudinal perspective within a pregnancy. Longitudinal analyses across periods of time and healthcare contacts allow the ability to include only managements based upon true conditions, ensuring that only appropriate and correct referrals are included in the calculation rather than all referrals. An ideal data source for complex indicators would be longitudinally collected at the point-of-care to minimize the need for post data-collection processing. This would ensure that both numerators and denominators were collected simultaneously, and mitigate issues from recall bias of either care providers or mothers. In addition, one of the largest criticisms of the Lives Saved Tool is the quality of estimates around maternal mortality. The current use of indirect estimates greatly increases the likely uncertainty around the LiST estimates of maternal mortality. These results should increase the validity and reliability of future analysis with such data, simply because fewer assumptions will be needed.

The paper-based routine health information systems in Palestine, as in many other places, rely on care providers identifying key characteristics about patients and reporting to district and national health authorities, who aggregate and process the data to generate national indicators. The validity of any individual diagnosis is unknown. This additional reporting burden on care providers limits the ability to demand reporting of a comprehensive set of clinical data, and thus results in a reporting system focused on only the highest priority indicators. Complex health conditions and reporting chains can lead to either over- or under-reporting. For example, knowing the number of women referred for diabetes is useful, but does not indicate the proportion of women correctly diagnosed with diabetes or appropriately referred, leaving the system unable to rectify underlying problems. To create more actionable indicators, providers would need to document every diabetes test, the number of women positive and the number referred according to recommended guidelines. This extensive task is not likely to be a valuable use of time in a paper-based system. The routine system in Palestine also relies on reporting by two different levels of clinics (primary and referral), which makes it difficult to ensure that women are correctly included only once, in either the numerator or denominator, potentially leading to biases. Routine reporting data should be limited and focused on critical indicators that cannot be collected easily in clinical data sources or those needed to triangulate with other sources.

Typically, the primary source of coverage data used in LiST is household survey data, such as the MICS presented here. However, very little data are available from these surveys to directly populate antenatal care (or childbirth care) indicators. Information on antenatal services received or antenatal care attendance can be used to indirectly calculate several other indicators. However, these estimates are dependent on maternal recall, which may be biased towards experiences of women with difficult pregnancies who would tend to remember care more completely relative to uneventful pregnancies and deliveries, while excluding pregnancies ending in stillbirth or miscarriage. Although these indirect indicators (together with non-antenatal care indicators) can be useful for planning, these surveys are typically conducted only every five years making their input less timely for shorter-term planning or course-correction. Additional questions should be asked about the utility of these indirect estimates (which were formulated with sub-Saharan African data) when compared to actual values extracted from antenatal care records. If the Kanyangarara formula is applied to the paper-based antenatal records and the eRegistry, respectively, approximately 62 and 61% of women are estimated to be correctly managed for hypertension while the clinical data indicated that only 7 and 10% were correctly managed. The differences were much smaller for the diabetes management indicator which were predicted to be 29 and 31% respectively, while the actual clinical values were 13 and 35%, respectively.

Data extracted from paper-based antenatal care records and the eRegistry contained the greatest quantity of data for direct analysis. They also allowed for computing indicators that most closely matched the ideals of the Lives Saved Tool (Table 3). Although differences in documentation may account for the different values reported, it should also be noted that indicators from the eRegistry document more carefully the details around management, which are not typically recorded in the paper records, and thus should theoretically be a more precise indicator of correct management. The simplified single checkbox of any iron-folate supplementation in paper records may have over-estimated current performance as the LiST analysis estimated more than two-fold higher numbers of anemia cases being averted in the eRegistry-based analysis compared with paper records. Assuming that care providers are correctly completing their documentation, these results should be more valid and more reliable than survey based data or routine reporting with the multiple additional layers of data processing required. They are certainly more direct estimates that have the potential to be more representative of facility care since they also include all pregnancies, not just all live-births.

Extracting data from paper-based records on a regular basis is neither feasible nor sustainable for routine monitoring due to the expense and tardiness of such a system, and without the quality assurance routines used in this study, also by the likelihood of transcription errors. In addition, paper records can be incomplete and do not have built-in validations at data entry, as seen with the tetanus toxoid vaccination data.

Although the development of an eRegistry is time-consuming and resource-intensive, and up-front implementation costs are relatively high, the benefits can be wide-ranging by integrating multiple digital health interventions in a single system. In Palestine, the point-of-care data entry currently serves as an interactive checklist with clinical decision support, with integrated audit and feedback components and a reminder system for pregnant women. On the back-end, the system routinely generates key indicators at national, sub-national and clinic levels without requiring burdensome reporting.

A limitation of the eRegistry system in Palestine is that it is currently only available in public sector facilities and does not include private or non-governmental organization (NGO) facilities, nor public hospitals. Population coverage cannot be measured with the eRegistry data in this setting. Although the lack of data from private and NGO facilities does not affect the analysis of care delivered at public facilities, LiST analysis might predict larger health improvements than actually could occur, due to missing data on referred patients who seek care in external facilities. At the same time, population based surveys can provide the data needed to understand the flow of patients between public and private or NGO sectors and thus act as a calibration of the clinical data, in conjunction with routine reported data. The lack of data from any of the hospitals also limited the ability to define the interventions in terms of full quality of care at referral centers. However, it is likely that adding this information would only decrease the proportion of women correctly managed.

## Conclusions

This study has clearly demonstrated the notable variability of information available for decision making based on the data source chosen in Palestine. Selection of the most complete and appropriate data source for policy and planning is critical. Many frameworks have been developed that attempt to characterize the features of priority setting and networks for informing policy decisions [21–24]. Studies have evaluated the barriers and facilitators to evidence-based decision making at national and local levels, and systematic reviews have described systems for incorporating research evidence into decision-making and less frequently, described the utilization of burden of disease data in decision-making [25, 26]. This paper feeds the discussion around how to support evidence informed decision-making at national levels by outlining the pros and cons of various data sources. We demonstrated the significant data driven benefits for the health system from utilizing data automatically extracted from a digital registry of health contacts – both in terms of quantity and quality. These distal benefits of an eRegistry along with more immediate clinical benefits to care providers and clients can also be used to inform a cost-benefit analysis for implementing complex health system interventions.

## Additional files

Additional file 1: Calculations for calculating coverage with the Kanyangarara method This file contains the raw data used and resultant coverage data generated when using the Kanyangarara method. (XLSX 9 kb)

Additional file 2: Detailed calculations of coverage indicators from paper records and from the eRegistry. This file contains the detailed and stepwise calculations of the hypertension, pre-eclampsia and diabetes coverage indicators calculated from both the paper records and the eRegistry. (XLSX 52 kb)

## Abbreviations

ANC: Antenatal care
DHS: Demographic and Health Survey
LiST: Lives Saved Tool
MICS: Multiple indicator cluster survey
MMR: Maternal mortality ratio
NGO: Non-governmental organization
